# An Overview of the Deubiquitinase USP53: A Promising Diagnostic Marker and Therapeutic Target

**DOI:** 10.2174/0113892037292440240518194922

**Published:** 2024-06-03

**Authors:** Guangce Xia, Yulin Guo, Jiajia Zhang, Meng Han, Xiangchao Meng, Ji Lv

**Affiliations:** 1 First College of Clinical Medicine, Hebei North University, Zhangjiakou 075000, China;; 2 First Hospital of Qinhuangdao Affiliated to Hebei North University, Qinhuangdao 066000, P.R. China;; 3 Breast Disease Diagnosis and Treatment Center, First Hospital of Qinhuangdao, Qinhuangdao, Hebei Province 066000, P.R. China

**Keywords:** USP53, deubiquitinase, ubiquitination, diagnostic marker, therapeutic target, bioinformatics

## Abstract

Ubiquitination and deubiquitination are important mechanisms to maintain normal physiological activities, and their disorders or imbalances can lead to various diseases. As a subgroup of deubiquitinases (DUBs), the ubiquitin-specific peptidase (USP) family is closely related to many biological processes. USP53, one of the family members, is widely expressed in human tissues and participates in a variety of life activities, such as cell apoptosis, nerve transmission, and bone remodeling. Mutations in the USP53 gene can cause cholestasis and deafness and may also be a potential cause of schizophrenia. Knockout of USP53 can alleviate neuropathic pain induced by chronic constriction injury. Loss of USP53 up-regulates RANKL expression, promotes the cytogenesis and functional activity of osteoclasts, and triggers osteodestructive diseases. USP53 plays a tumor-suppressive role in lung cancer, renal clear cell carcinoma, colorectal cancer, liver cancer, and esophageal cancer but reduces the radiosensitivity of cervical cancer and esophageal cancer to induce radioresistance. Through the in-depth combination of literature and bioinformatics, this review suggested that USP53 may be a good potential biomarker or therapeutic target for diseases.

## 1 INTRODUCTION

### 1.1 Ubiquitination: A Post-Translational Modification

Gene expression is regulated at multiple levels, including transcriptional, post-transcriptional, translational, and post-translational ones [1-4]. Among them, post-translational modifications are the most abundant and diversified, such as phosphorylation, methylation, glycosylation, acetylation, and ubiquitination, which can affect the localization, stability, activity, and function of proteins [5-7]. Among all of them, ubiquitination is the most widely studied.

Ubiquitination is a post-translational modification of proteins that regulates the stability and activity of target proteins by covalently linking ubiquitin (a small protein containing 76 amino acids, 8.5 kDa) to the target protein [8, 9]. The ubiquitination process involves the stepwise synergistic action of three enzymes: the ubiquitin-activating enzyme (E1), which activates ubiquitin molecules in the presence of ATP, then transmits the activated ubiquitin molecules to the ubiquitin-conjugating enzyme (E2), and finally, the ubiquitin ligase (E3), which links the E2-bound ubiquitin to the sub strate protein. E3 is highly specific for its substrate and is, therefore, the key enzyme in the entire ubiquitination process [9-11]. Ultimately, the proteasome breaks down ubiquitin-tagged proteins into peptides, amino acids, and reusable ubiquitin. The ubiquitin-proteasome system is involved in the degradation of the vast majority of human proteins [11].

### 1.2 Deubiquitination: Reversal of Ubiquitination

**Fig. (1):**
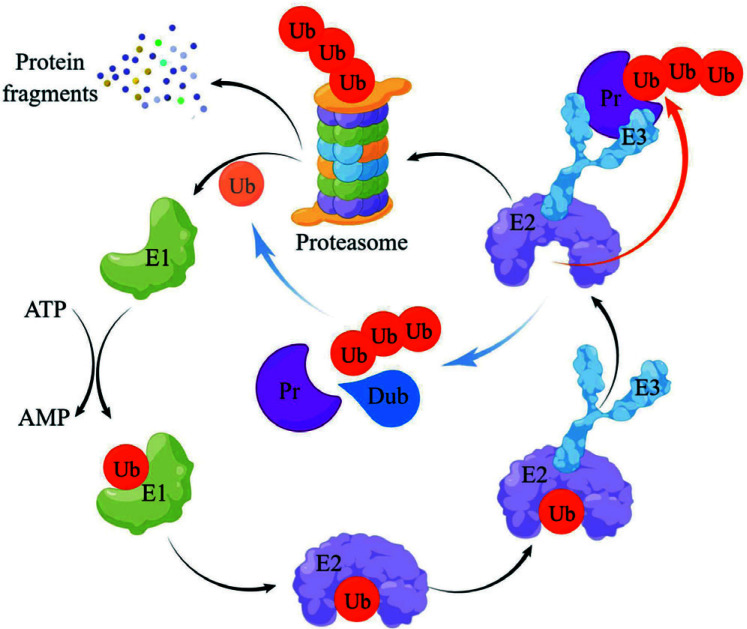
Biological process of ubiquitination and deubiquitination: their interaction maintains the homeostasis of proteins and free ubiquitin in the organism.

Deubiquitination is the reverse reaction of ubiquitination, which affects the function and fate of the target protein by removing ubiquitin from the target protein [12]. The process of deubiquitination mainly relies on DUBs to cleave ubiquitin and inactive ubiquitin precursors on target proteins to maintain the free ubiquitin pool and protein degradation rate *in vivo* [13, 14]. More than one hundred DUBs have been identified to date, including nine subgroups: USPs, herpesvirus tegument USPs (htUSPs), Machado-Josephine domain proteases (MJDs), ovarian tumor proteases (OTUs), monocyte chemoattractant protein-inducible proteins (MCPIPs), ubiquitin carboxy-terminal hydrolases (UCHs), zinc finger-containing ubiquitin peptidases (ZUPs), motif interacting with ubiquitin-containing novel DUB family (MINDYs), and JAM/MPN domain-related Zn-dependent metalloproteinases (JAMMs) [13, 15-17]. Among them, JAMMs are metal-dependent proteases, while the remaining families belong to serine/cysteine proteases. The presence and activity of these enzymes are important for the biological behavior of almost all cells [18]. There is a mutual regulatory relationship between ubiquitination and deubiquitination, and they jointly maintain the dynamic balance of protein action levels [12] (Fig. **1** by Figdraw).

### 1.3 Roles of Ubiquitination and Deubiquitination

Normally, ubiquitination leads to degradation or functional changes in the target protein, while deubiquitination can reverse these effects and restore the stability and function of the target protein [12, 19]. They play important roles in many biological processes, such as cell cycle regulation, gene damage repair, cell signal transduction, protein interaction network, and quality control [19-21]. Some factors, such as genetic and epigenetic changes, can lead to the disorder of ubiquitination and deubiquitination [21]. The disorder of their balance is closely related to the occurrence and development of a variety of diseases, such as tumors, neurodegenerative diseases, and autoimmune diseases [22-24]. Understanding the underlying mechanisms may provide new therapeutic directions for these diseases.

For this purpose, literature was retrieved and downloaded from Pubmed (https://pubmed.ncbi.nlm.nih.gov/) and CNKI (https://www.cnki.net/) with ubiquitination, deubiquitination, USPs, and USP53 as keywords, respectively, then the best representative ones were selected for intensive reading. Combining biological information from public databases and websites, this study eventually presented a comprehensive review of the current research progress in the deubiquitinase USP53 in order to lay a foundation for subsequent research.

## 2 USP FAMILY AT A GLANCE

**Fig. (2):**
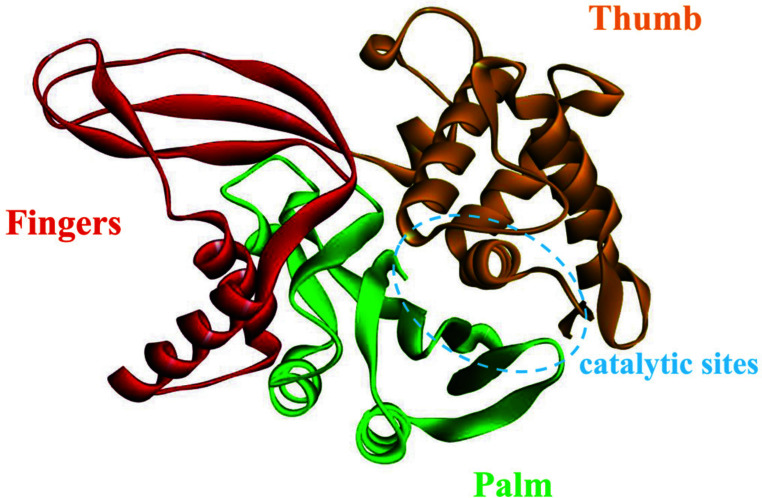
USP domain: It looks like an open right hand.

With a total of 58 known members, USPs constitute the largest and most functionally complex family of DUBs [25, 26]. All the family members have a highly conserved USP domain of about 300-800 amino acids, resembling an unfolded right hand, including the palm, thumb, and finger subdomains [26, 27] (Fig. **2** by Discovery Studio). The catalytic sites of USP, a specific catalytic area consisting of cysteine, histidine, and aspartic acid, exist between the palm and thumb, and the finger structure is responsible for the connection and interaction with ubiquitin [27-29]. This is a typical conformation and common feature of USPs, which can promote their transition from an inactive state to a certain catalytic activity upon binding to ubiquitin [27, 28]. Additionally, they exhibit relative structural diversity, showing additional domains and terminal extensions, such as domains specific to USP (DUSP), zinc finger ubiquitin-binding domain (ZnF-UBP), ubiquitin-interacting motif (UIM), ubiquitin-like domain (UBL), ubiquitin-associated (UBA), *etc.* [26, 30]. These different domains play a role in functional specificity.

As the major DUB members, USPs are closely related to many biological processes, mainly involving:

### 2.1 Regulation of Cell Cycle

USP2 stabilizes cyclin D1 and promotes cell cycle progression [31]. Knockdown of USP3 decreases the level of CDC25A phosphatase, resulting in cell cycle delay [32]. USP22 regulates Cyclin B1, and its knockdown causes G2/M phase arrest [33]. USP39 stabilizes CDK1 and cyclin B1, thereby driving cell cycle progression [34].

### 2.2 Involved in Apoptosis

USP2 and USP7 can stabilize MDM4 and MDM2 to degrade p53, respectively, leading to an anti-apoptotic phenotype [35, 36]. Overexpression of USP24 stabilizes Securin and Bax to induce apoptosis [37]. USP27X can stabilize BCL2L11 to enhance the anti-apoptotic effect of MAPK [38]. USP30 inhibits apoptosis by stabilizing Parkin [39].

### 2.3 Regulation of Signaling Pathways

USP4 negatively regulates Wnt by interacting with Nemo-like kinases [40]. USP18 targets NEMO and TAK1 to inhibit the NF-κB pathway [41]. Moreover, USP21 could stabilize IL33 and enhance NF-κB signal transduction [41]. USP8, USP17, and USP18 can stabilize EGFR levels by deubiquitination and regulation of subcellular localization [26].

### 2.4 DNA Damage Repair

USP4 binds CtIP and MRN complexes to regulate DDR and thereby promotes homologous recombination [42]. USP7 plays an important role in DNA repair by deubiquitinating XPC, a key recognition factor of DNA damage, to avoid its degradation [43]. USP34 is responsible for stabilizing the E3 ubiquitin ligase RNF138 in response to DNA damage [44].

It has been found that the USP family is also correlated with various diseases, including metabolic diseases, immune diseases, and neurodegenerative diseases, especially malignant tumors [26, 45]. Hiroshi has pointed out that USP1 is involved in DNA damage-related islet β-cell apoptosis in patients with type 2 diabetes, whereas USP22 plays a protective role in β-cell dysfunction [30]. USP7 is highly active in Treg cells, and depletion of USP7 in Treg cells in inflammatory bowel disease models deprives them of their ability to resolve inflammation, suggesting a role for USP7 in adaptive immunity [46]. In addition, the expression of USP7 is increased during myocardial ischemia/reperfusion injury in mice, while it inhibits the production of oxygen free radicals and myocardial cell apoptosis, reduces myocardial tissue damage, and improves cardiac function [47]. USP13 inhibitors reduce the burden of neurotoxic proteins in neurodegenerative diseases by antagonizing deubiquitination [48].

### 2.5 In Terms of Cancer Research

USP2 is involved in the activation of the c-Myc pathway and regulates prostate cancer formation [49]. USP7 promotes the growth of non-small cell lung cancer cells by stabilizing Ki-67 protein, while metformin can inhibit the proliferation of esophageal cancer by upregulating USP7, suggesting that USP7 plays different roles in different types of tumors [50-52], USP4 and USP15 can stabilize TGF-β receptors and increase TGF-β-mediated EMT, leading to metastasis of hepatocellular carcinoma and glioblastoma [53-55]. Furthermore, USP22 promotes the proliferation, migration, and invasion of pancreatic, breast, gastric, and colorectal cancers and is associated with poor prognosis [56-59]. USP39 down-regulates the p53/p21 signaling pathway and stabilizes CDK1 and cyclin B1 to promote the proliferation of ovarian cancer cells [34]. In recent years, a growing number of researchers have paid attention to the potential of USPs as cross-disciplinary therapeutic targets, and many USPs have been targeted for the development of inhibitors [13, 28]. Therefore, further research and exploration of these will provide important theoretical and practical basis for disease treatment.

## 3 STRUCTURE AND PHYSIOLOGICAL FUNCTIONS OF USP53

USP53 was originally called KIAA1350, which was discovered and named when screening the human genome database in 2003. It is located on chromosome 4, with a sequence of 3280 bases, encoding 1073 amino acids, with the protein being about 120 kda in size [60, 61].

**Fig. (3):**
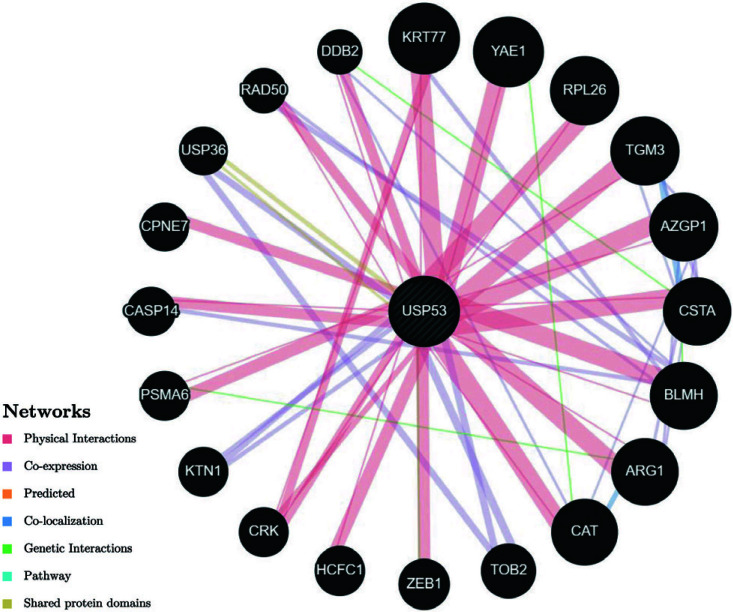
The network of interactions between USP53 and other genes.

The structure of USP53, despite the presence of the catalytic triad as well, lacks an essential histidine and thus belongs to the non-protease homolog of the USP family [27, 62]. For such a USP without protease activity, USP53 is not only closely related to ubiquitin and ubiquitin conjugates but also interacts with other proteins involved in signal transduction, substance metabolism, or other functions [63, 64] (Fig. **3**, http://genemania.org/). For example, USP53 acts as a tight junction protein that can interact with TJP1 and TJP2, which are also tight junction proteins [60]. Preliminary analysis revealed that USP53 has a variety of post-translational modifications, including phosphorylation of serine, threonine and tyrosine residues (S22, S95, S368, S510, S560, S562, S563, S572, S798, S839, S848, T1046, T1048, Y1049, 1073), arginine methylation (R328), ubiquitination of two residues (K231 and S576 in non-functional catalytic domains) and lysine acetylation (K578), which have not yet been studied in detail [65, 66]. Three double cysteine motifs in the structure of USP53 were indicated by bioinformatics, as shown in Fig. (**4**), which may be its binding sites. (https://prosite.expasy.org/).

**Fig. (5):**
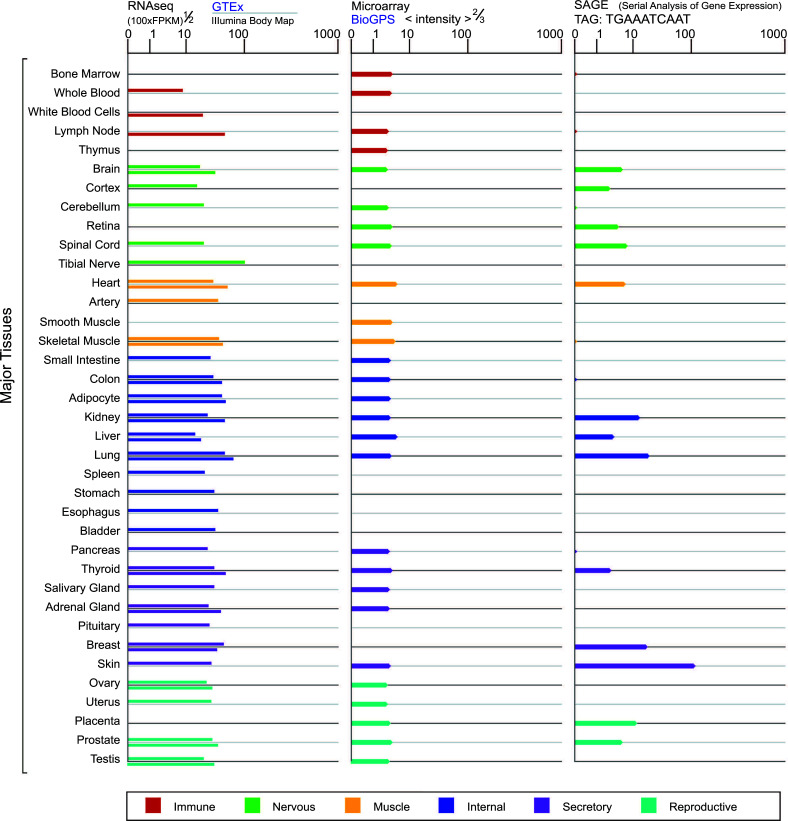
Distribution of USP53 in human tissues.

USP53 homologs exist in the genome of all vertebrates, widely expressing in the liver, kidney, digestive tract, lung, inner ear, fat, bone, and brain tissues, and are involved in life activities, such as cell apoptosis, glycolysis, nerve transmission, fat metabolism, bone formation, and bone homeostasis (Fig. **5**, https://www.genecards.org/). However, only a few specialized studies have been conducted so far [61, 62, 67, 68].

## 4 RELATIONSHIP BETWEEN USP53 AND DISEASES

### 4.1 Role of USP53 in Cholestasis and Deafness

Hereditary cholestasis is a group of autosomal recessive inherited heterogeneous liver diseases [69]. The main clinical manifestations include intrahepatic cholestasis, pruritus, and jaundice, and some are accompanied by deafness [69, 70]. It has been reported that patients with USP53 biallelic mutations developed normal or low gamma-glutamyl-transferase (GGT) cholestatic liver disease [70-73]. Study data suggested that individuals with this mutation could exhibit different phenotypes and that hearing loss was not present in all cases [73-75].

USP53 is expressed in cochlear hair cell and sertoli cell subsets, binding to the C-terminal S long tail of TJP1 and TJP2, co-localizing to and interacting with tight junctions in the stria vascularis, and regulates barrier properties and mechanical stability of tight junctions, which are essential for the survival of auditory hair cells and the stability of the inner ear environment [60, 76]. Marcin *et al.* reported the phenotypic characteristics of a mutant USP53 allele (known as mambo), which can be induced in mice using nitrocarbamide to mutate Cys228 to serine, leading to degeneration of cochlear hair cells, destruction of the organ of Cortis, and rapid progressive hearing loss [60]. USP53 is a novel tight junction component required for maintaining the integrity of the auditory system.

### 4.2 Role of USP53 in Neurological and Psychiatric Disorders

Neuropathic pain, characterized by allergy, hypersensitivity, secondary pain, and spontaneous pain, is considered to be a hypersensitive response to various stimuli caused by primary injury and dysfunction of the somatic nervous system [77, 78]. Li *et al.* reported that the expression of USP53 was up-regulated in the brain and spinal cord tissues of SD rats with chronic compressive injury, and knockout of the USP53 gene could alleviate neuropathic pain and inhibit inflammatory response in this model rats [79]. Their study demonstrated that USP53 aggravated chronic compressive injury-induced neuropathic pain by activating the FKBP51/RhoA/ROCK signaling pathway.

José *et al.* identified a variant in USP53 (p.Cys228Arg) as an underlying cause of schizophrenia [66]. Although such a kind of variant is rare in databases, the genetics of mental disorders are complex, ranging from common predisposition variants with modest phenotypic effects to rare deleterious mutations with substantial effects. Cysteine residues in USP53 mambo mutations were found to correspond to those in patients with schizophrenia [60]. USP53 has been implicated in several pathways related to schizophrenia, such as ubiquitin processing, folate metabolism, and tight junction physiology [80, 81]. It may be the first USP directly associated with psychiatric disorders.

### 4.3 Role of USP53 in Bone Diseases

In bone, the synchronous activity of osteoblasts and osteoclasts maintains bone homeostasis and integrity [82]. Therefore, it is crucial to investigate the molecular mechanisms controlling bone formation and bone homeostasis to help understand the underlying biological principles of bone diseases. RANKL is a major regulator of osteogenesis, activating osteoclasts and promoting osteolysis. Overexpression of RANKL can lead to a series of bone-destructive diseases, such as osteoporosis, rheumatoid arthritis, and cancer bone metastasis [61, 82]. It has been found that USP53 deficiency upregulates RANKL expression in osteoblasts and bone marrow adipocytes to support osteoclast production through the VDR-SMAD3 pathway and promotes bone marrow adipogenesis [61].

USP53-deficient mice had a low bone mass phenotype, with decreased trabecular bone index and cortical bone parameters, impaired bone strength and mechanical properties, as well as a significant increase in bone marrow adipose tissue volume [61, 83]. This was not only similar to the skeletal changes associated with aberrant USP53 expression in Cantu syndrome but also consistent with the down-regulation of USP53 expression in the bone marrow of osteoporosis patients [84, 85]. It has been found that USP53 was a target of the PTH-NACA pathway in osteoblasts and a regulator of mesenchymal stem cell differentiation [83]. Baek *et al.* also confirmed the role of USP53 in regulating the osteogenic differentiation of human bone marrow-derived mesenchymal stromal cells [85].

In conclusion, USP53 is a new regulator of bone development, which can maintain bone homeostasis through osteoblasts and osteoclasts. Its mutation or abnormal expression may be closely related to the occurrence and development of a variety of bone diseases.

### 4.4 Role of USP53 in Malignant Tumors

Although the role of USPs in cancer has been increasingly recognized by scholars, the function and specific mechanism of USP53 in malignant tumors have been lacking corresponding research, which needs to be further explored.

#### 4.4.1 USP53 in Lung Cancer

Lung cancer is one of the most common malignant tumors and the leading cause of cancer death worldwide [86]. Studies have shown that USP53 expression was down-regulated in lung cancer, which was significantly lower than that in normal adjacent tissues and was related to tumor size, smoking status, and lymph node metastasis. There was a statistical difference in the survival rate of patients with high and low expression of USP53, which was a prognostic protective factor for lung cancer [67, 87].

Zhao *et al.* found that the expression of USP53 in lung cancer tissues was positively correlated with the expression of FKBP51 while negatively correlated with the expression of p-AKT1 [67]. Serine/threonine protein kinase (AKT) is involved in regulating a variety of cellular processes, including survival, migration, proliferation, glucose metabolism, and protein synthesis [88]. FKBP51 has been shown to participate in apoptosis and cisplatin sensitivity of lung adenocarcinoma cells through the p53 signaling pathway [89]. USP53 deubiquitinated FKBP51 in lung adenocarcinoma cells (H1975 and HCC827), which, in turn, dephosphorylated AKT1, inducing cell apoptosis and inhibiting glycolysis through this signaling pathway. Moreover, animal experiments demonstrated that USP53 overexpression inhibited tumor growth in nude mice [67]. ZEB1 is closely related to epithelial-mesenchymal transformation (EMT), invasion, metastasis, and chemotherapy resistance of cancer cells [90]. In another study, Western Blot, MTT, transcriptome sequencing, transwell, and ubiquitination experiments demonstrated that USP53 can regulate the expression of ZEB1 in lung cancer cells (A549 and H1975), thereby inhibiting EMT and further reducing cell migration, invasion, and cisplatin tolerance [87].

#### 4.4.2 USP53 in Clear Cell Renal Cell Carcinoma (ccRCC)

Renal cell carcinoma is the most lethal malignant tumor in the urinary system, with ccRCC being the major histological subtype, accounting for about 75% and causing more than 140,000 related deaths each year [91].

Ubiquitination-deubiquitination homeostasis is a vital factor in the occurrence and development of ccRCC. Gui reported that USP53 was down-regulated in ccRCC tissues, and patients with high expression can achieve better survival outcomes [92]. USP53 inhibited the proliferation and metastasis of ccRCC cells (786-O and Caki-1) *via* the NF-κB pathway *in vitro*, and the knockdown of USP53 promoted the growth of transplanted tumors in nude mice with ccRCC. NF-κB plays an important role in immune and inflammatory responses and regulates various biological processes, such as cell stress, proliferation, apoptosis, angiogenesis, and cancer metastasis [93]. USP53 can inhibit the complex of p50 and p65 by deubiquitinizing IκBα, thereby inhibiting the activity of the NF-κB pathway and ultimately inhibiting the proliferation and metastasis of ccRCC [92]. These findings contribute to a better understanding of the pathogenesis of ccRCC and provide a new potential therapeutic target for it.

#### 4.4.3 USP53 in Cervical Cancer

Cervical cancer is the most common gynecological malignant tumor, and more than half of the patients receive clinical radiotherapy, the efficacy of which, however, varies greatly from individual to individual, and some patients show obvious radiotherapy resistance [94].

Zhou *et al.* found, by means of histopathological and cytofunctional tests, that USP53 was related to the radiotherapy effect of cervical cancer, and it can enhance the radioresistance of cervical squamous cells [95]. Knockdown of USP53 followed by irradiation can cause G2/M phase arrest and inhibit DNA damage repair of human SiHa cells, resulting in a decreased cell survival rate. In these processes, the cell cycle arrest occurred by regulating CDK1 and Chk2, while the inhibition of DNA damage repair was achieved by DDB2 regulation.

#### 4.4.4 USP53 in Colorectal Cancer

Colorectal cancer is a common malignant tumor of the digestive tract, which increasingly affects the younger population, and the survival of advanced cases has not been optimistic in recent years [96].

Through bioinformatics analysis, Real-Time PCR, and immunohistochemistry, Shen found that USP53 was significantly down-regulated in colorectal cancer tissues, and its expression was negatively correlated with tumor size while positively correlated with chemotherapy response and prognosis of patients [97]. CCK8 and clonal formation experiments suggested that overexpression of USP53 can inhibit the proliferation and clonogenesis of HCT116 colorectal cancer cells *in vitro*. The role of USP53 in the diagnosis and treatment of colorectal cancer deserves further exploration.

#### 4.4.5 USP53 in Hepatocellular Carcinoma (HCC)

HCC accounts for about 75-85% of primary liver cancers, and despite great advances in treatment, patients still have a high recurrence rate and a poor prognosis [98].

Yao *et al.* used immunohistochemistry, Western Blot, and bioinformatics analysis to find that USP53 expression was down-regulated in HCC, which was related to the high degree of malignity and poor prognosis of patients [99]. In their research, USP53, as a tumor suppressor, inhibited the proliferation and migration of Huh-7 and HCCLM3 cells *in vitro*, promoting apoptosis by deubiquitinating and stabilizing cytochrome C (a key apoptotic protein) to prolong its active time and restrained the growth of transplanted tumor *in vivo*. This study may provide a new strategy for the HCC treatment.

#### 4.4.6 USP53 in Esophageal Cancer (ESCA)

ESCA is one of the most lethal malignant tumors of the digestive system, with an average 5-year overall survival rate of 15%, causing about 400,000 deaths each year [100].

Cheng *et al.* reported that USP53 expression was down-regulated in ESCA, and patients with low expression have worse survival outcomes [101]. Overexpression of USP53 inhibited the growth of ESCA *in vitro* and *in vivo*, while its knockdown exerted the opposite effect, which, in turn, could be reversed by an AMP-activated protein kinase inhibitor. USP53 expression in ESCA can be increased by H3K27 acetylation combined with its promoter region. In addition, USP53 also suppressed glycolysis, oxidative metabolism, and mitochondrial dynamics. This study finally revealed that USP53 was activated by H3K27 acetylation in a variety of ESCA cells, whereafter suppressing proliferation, promoting apoptosis, and inducing mitochondrial damage by inactivating the AMPK pathway.

Radiotherapy is one of the main treatments for ESCA, but some patients are susceptible to radiotherapy resistance. Nrf-2 is an important antioxidant protein in the human body, which can reduce the sensitivity of cells to ionizing radiation [102]. Tian *et al.* confirmed by Real-Time PCR, Western Blot, CCK8, and clonal formation experiments that USP53 can increase the expression of Nrf-2 in Eca109 cells and then activate the expression of downstream anti-oxidative stress genes HO-1 and NQO-1, resulting in radioresistance of the cells [103]. However, the roles of USP53 in ESCA need to be further explored.

## CONCLUSION

This study comprehensively reviews the latest research progress in USP53 in different fields. In general, the current research on this topic is not extensive enough, and the specific mechanism of USP53 in physiology and disease is still lacking deep exploration. For example, USP53 can exert proliferation-inhibiting and pro-apoptotic effects on ESCA cells *in vivo* and *in vitro* but also induce radiotherapy resistance, suggesting that the impact of USP53 on ESCA patients receiving radiotherapy needs to be further explored. It is hoped that future studies can clearly reveal the detailed role of USP53 and its mechanism in the human body and provide great help for medical progress and people's health.

## Figures and Tables

**Fig. (4):**
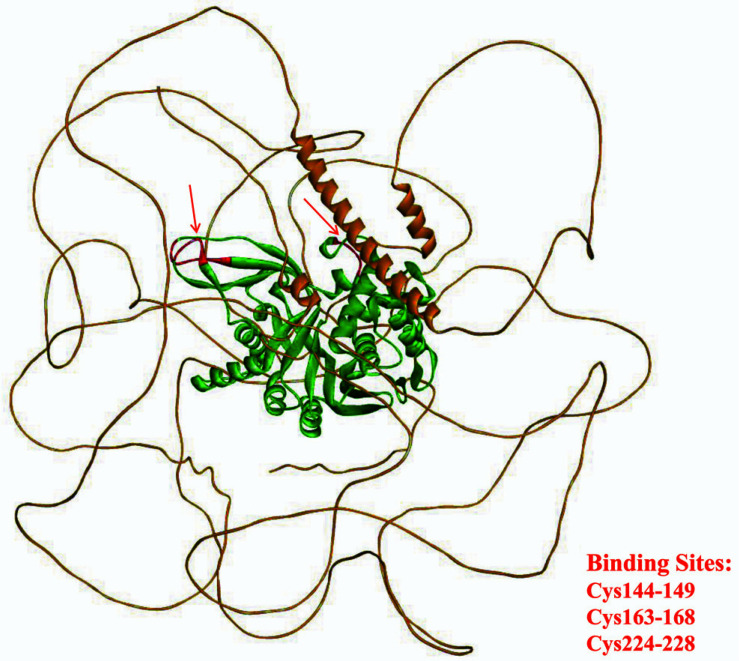
Structure of USP53: the predicted binding sites are shown in red.

## LIST OF ABBREVIATIONS

USP53: Ubiquitin-specific Peptidase 53
DUBs: Deubiquitinases
htUSPs: Herpesvirus Tegument USPs
MJDs: Machado-Josephine Domain Proteases
OTUs: Ovarian Tumor Proteases
MCPIPs: Monocyte Chemoattractant Protein-inducible Proteins
UCHs: Ubiquitin Carboxy-terminal Hydrolases
ZUPs: Zinc Finger-containing Ubiquitin Peptidases
MINDYs: Motif Interacting with Ubiquitin-containing Novel Dub Family
JAMMs: JAM/MPN Domain-related Zn-dependent Metalloproteinases
ZnF-UBP: Zinc Finger Ubiquitin-binding Domain
UIM: Ubiquitin-interacting Motif
UBL: Ubiquitin-like Domain
UBA: Ubiquitin-associated
MAPK: Mitogen-activated Protein Kinase
ccRCC: Clear Cell Renal Cell Carcinoma
HCC: Hepatocellular Carcinoma
ESCA: Esophageal Cancer
